# Practical strategies to achieve resilient health systems: results from a scoping review

**DOI:** 10.1186/s12913-024-10650-8

**Published:** 2024-03-06

**Authors:** David Bishai, Basma M. Saleh, Maryam Huda, Eman Mohammed Aly, Marwa Hafiz, Ali Ardalan, Awad Mataria

**Affiliations:** 1 University of Hong Kong School of Public Health, Hong Kong, China; 2 Institute of Global Health and Human Ecology, American University of Cairo, Cairo, Egypt; 3 Department of Community Medicine, Aga Khan University, Karachi, Pakistan; 4 World Health Organization Eastern Mediterranean Regional Office, Cairo, Egypt

**Keywords:** Resilience, Health systems, Public health, Performance improvement

## Abstract

**Background:**

This paper presents the results of a systematic review to identify practical strategies to create the institutions, skills, values, and norms that will improve health systems resilience.

**Methods:**

A PRISMA 2020 compliant systematic review identified peer-reviewed and gray literature on practical strategies to make health systems more resilient. Investigators screened 970 papers to identify 65 English language papers published since 2015.

**Results:**

Practical strategies focus efforts on system changes to improve a health system’s resilience components of *collective knowing, collective thinking, and collaborative doing.* The most helpful studies identified potential lead organizations to serve as the stewards of resilience improvement, and these were commonly in national and local departments of public health. Papers on practical strategies suggested possible measurement tools to benchmark resilience components in efforts to focus on performance improvement and ways to sustain their use. Essential Public Health Function (EPHF) measurement and improvement tools are well-aligned to the resilience agenda. The field of health systems resilience lacks empirical trials linking resilience improvement interventions to outcomes.

**Conclusions:**

The rigorous assessment of practical strategies to improve resilience based on cycles of measurement should be a high priority.

**Supplementary Information:**

The online version contains supplementary material available at 10.1186/s12913-024-10650-8.

## Background

COVID-19 highlighted overconfidence in health system resilience [1]. Resilience includes efforts to learn from a crisis and transform the health system on an ongoing basis [2]. Several sources have converged to recommend that achieving resilience in health security requires better governance, leadership, financing, and equity [3].

WHO has outlined a vision placing primary health care as the foundation for dual goals of health security and universal health coverage [3, 4]. WHO’s “resilience toolkit” has assembled close to 100 products to support efforts to build resilience [5]. It remains unclear how resilience tools can actually be applied and how to sustain the use of these tools. The resilience agenda needs an evidence base of well-defined actions. There is a window of attentiveness among citizens and leaders inside and outside the health sector adding urgency to what was already a strong rationale to accelerate the implementation of resilience strategies [6].

A recent systematic review of empirical work on resilience noted an imbalance between theoretical understanding of resilience and practical efforts to apply these concepts [7]. Many policy makers and health system leaders face obstacles in designing policies, programs, and budget allocations that increase resilience. The rationale for this systematic review is to examine current knowledge about how to put the concept of resilience into practice in health systems.

Health systems are complex collections of agents and units governed by institutions. They have variable success in adapting coherently towards an ultimate goal of better health. Sub-systems focus on canonical building blocks like financing, service delivery, supplies, etc. Health systems operate from micro, meso, to macro levels. Despite the sprawling landscape, the concept of resilience can be applied at all levels and in all subsystems. The resilience literature we review can be expected to come from practical efforts in various domains of the health system, and as long as it sheds light on how to implement resilience, it will be in scope.

This paper addresses the following question: How does an increase in health system resilience get put into practice? The objective is to gather answers to questions of who, what, where, when, why and how to improve resilience. The paper applies a systematic literature review about the implementation of resilience strategies to achieve health security in low- and middle-income countries.

Answers to “how to?” are circular and unhelpful if they end up using verbs like “strengthen”, “empower” or “invest in”. Planners and implementers need verbs like, “hire”, “purchase”, “legislate”, “measure”, “inform” and “meet with”. Meaningful contributions in the literature need to name who is to do what with whom, how, when, why, with a plan for accountability. We benefited from the Foroughi et al. (2022) framework to ask that resilience actions be classified according to their intermediate objectives, phases, and requirements [8].

## Methods

### Literature Review

A systematic literature review was conducted following PRISMA 2020 guidelines [9]. The search strategy was developed by starting with the term “resilience” and circumscribing it to the area of “health systems”. Because neither term is recognized by PubMed as a medical subject heading (MeSH), both terms were put in as field searches for title or abstract. Attempts to circumscribe this two-term search with other AND terms like “universal health coverage” or “health security” or “policy” became unacceptably restrictive. The final search terms used were: <(resilien*[Title/Abstract]) AND (“health syste*“[Title/Abstract])>. This search was confined to English language publications with publication dates after January 1, 2015. Literature databases included PubMed, Web of Science, and OAIster. The search was completed on October 25, 2022. Additionally, the project has examined the websites of relevant public health-related organizations (WHO Headquarters, WHO EMRO, Alliance for Health Policy and Systems Research, Health Systems Global, UNICEF, World Bank and CDC) in an attempt to identify articles and frameworks not indexed in the other databases.

The PubMed search produced 956 papers with 2 duplicates. Oaister and Web of Science contributed 1 paper each not identified by PubMed. Bibliographies in papers by Fridell (2020), Kuhlmannn (2021), WHO Toolkit (2022), and Alilio (2022), yielded an additional 16 citations that were not identified in the search databases [5, 10–12].

Rapid title screening was conducted by a single investigator to reduce the list to 136 titles which then underwent a second round of title screening by three investigators who narrowed the list to 87. Title-based screening excluded papers because they described resilience concepts that were outside the aims of this research, (e.g., resilience properties of whole mechanical systems or resilience of single organizations that did not extend to the health system). For example, articles were excluded if the article described resilience in contexts outside of the health systems context (e.g., armed conflict situations). We included documents if they described attempts to implement health system resilience or link it to universal health coverage or health security.

**Fig. 1:**
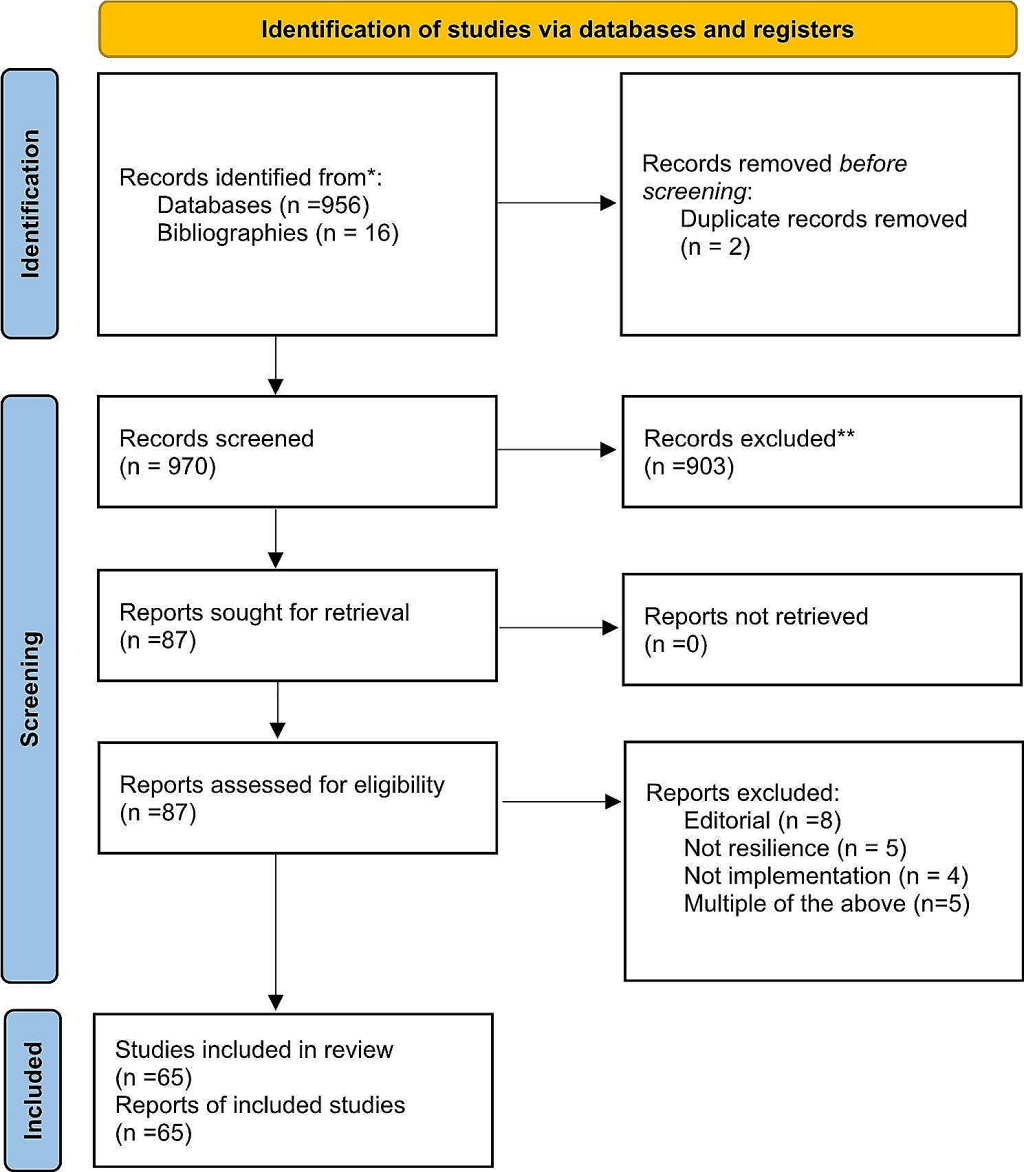
PRISMA diagram

The 87 that passed the title screening were then classified and further screened by a single investigator based on the abstract leading to an additional 22 exclusions. These abstract-based exclusions occurred when abstract review showed that they were editorials (e.g. introducing a special issue) or not about resilience, or not related to practical implementation of resilience. Finally, 65 papers were forwarded to the extraction stage. (See Fig. 1 PRISMA Diagram.)

All articles deemed relevant after title and abstract review were then read in their entirety by at least two members of the study team. A database was maintained in DistillerSR™ for each article’s contribution to the four key questions of the project: (1) Defining practical aspects of resilience; (2) Links to health security and UHC; (3) Resilience in practice; (4) Implementation. Extracted data were reviewed for recurrent themes related to the main question of practical strategies that can improve resilience. Despite the effort to screen out editorials based on abstract review, upon examining the full documents, it emerged that 16 papers were opinion, commentary, and expert advice and flagged as such.

## Results

The research themes in the extracted summaries were coded inductively using codes that emerged from the extracted texts (See Table 1).

**Table 1:** Coding System for Data Extracted from Included Studies

Code (Number of Papers)	Refers to
Defines Resilience (4)	Synthesizes an original resilience definition
Essential Public Health Functions (EPHF) (8)	How EPHF is a path to resilience
Everyday Resilience (9)	Pointing out overlap between everyday resilience and crisis resilience
Fragmentation (2)	Flags the problem of multiple vertical programs and levels of authority
Measure Resilience (8)	Focus on role of measurement
Multisectorality (4)	Need to connect health sector to all sectors
PHC (5)	Suggestions flagging a robust capability to offer PHC
Social inputs (3)	Suggestions about pre-crisis social capital, community, engagement
Supplies (1)	Need to build logistics and supply support
To Do/Gaps (1)	Gaps in the resilience agenda
Trust (6)	Pointing out the cycle of repeated positive interactions with trust as a path to resilience as Aware, Diverse, Self-regulating, Integrated, Adaptive
Who does what (14)	Suggestions about which workers need to be involved

We found fourteen papers specifying resilience strategies in terms of “who does what”. Other common themes pointed out the overlap between everyday resilience and crisis resilience (nine papers) and laid out approaches to measure resilience as a way to improve the governance, workforce capability, and cross-system coherence (eight papers).

**Fig. 2:**
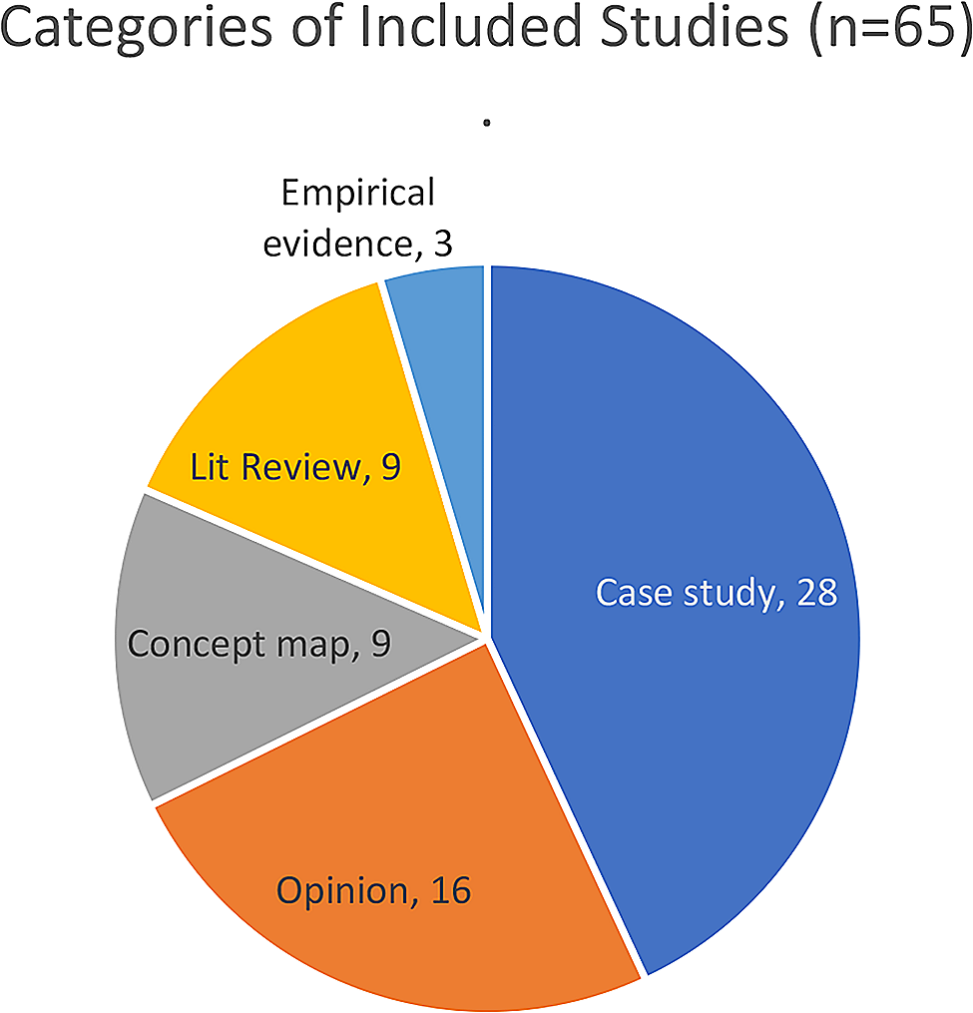
Categories of included studies

Other important themes were about how community trust, multisectoral engagement and social capital could be leveraged to improve resilience (See Table 1). The categories of included studies are shown in Fig. 2.

## Features of the resilience concept that affect implementation

Several recent literature reviews have focused on conceptual definitions [10, 13, 14, 15, 7]. Biddle et al. reviewed 71 papers on the topic from 2008 to 2019, but over half of the papers were published from 2017 to 2019 [7]. Ten of the papers reviewed by Biddle et al. have “Ebola” in the title, reflecting how the 2014 outbreak had triggered interest. However, resilience had earlier become a major focus of the Rockefeller Foundation during the presidency of Dr. Judith Rodin whose 2014 book *The Resilience Dividend* sparked popular attention through narrative case studies of community resilience under a variety of crisis situations [16]. Rodin’s book popularized a set of five pillars of community resilience as Awareness, Diversity, Self-Regulation, Integration, and Adaptation (A, D, SR, I, A) that figure heavily in later conceptual maps of resilient health systems [17, 18].

As more scholarship on the concept of health system resilience developed, related definitions echoed Rodin’s contribution that the concept of resilience was highlighted during times of crisis and that resilience involved an anticipatory practice of drawing together diverse strands of situational knowledge to deliberately build institutions that were responsive. One can see a refinement from Rodin’s original 5 pillars (A, D, SR, I, A) to the three most conserved elements of the resilience concept: Awareness, Self-Regulation, and Adaptation (A, SR, A). It is not that diversity and integration are unnecessary, but that they are subsumed if there is to be any true success with awareness, self-regulation, and adaptation. The refinement from five resilience elements to three is most obvious when Blanchet and co-authors note that resilience of a health system is, “its capacity to absorb, adapt and transform when exposed to a shock such as a pandemic, natural disaster or armed conflict and still retain the same control over its structure and functions.” [2] One synthesis of the three preserved elements of resilience that we found in the concept papers is an agreement that resilience is a form of intelligence. Intelligence implies that information is taken in, processed, and acted on [16, 17, 19, 22]. (And as per Rodin, to do this well, one would do it with respect to diversity and the ability to integrate). Intelligent systems -living or artificial- adapt to their situations by starting with afferent “sensing”, followed by “deliberating” either by unitary or social deliberation, and finally launch efferent “actions” that act upon the internal or external state.

Part of the attraction of the term “resilience” comes from its ambiguity. Its lack of clarity invites people from both politics and science to a crossroads area where the term “resilience” can be stretched to fit divergent goals and diverse perspectives [2]. Those who write about resilience typically feel free to adapt the term in various ways. Having a “big tent” word for what is desired from a health system is a gateway for the necessary multi-stakeholder, multi-perspective conversations that enable progress. Usefully ambiguous buzzwords can play an important galvanizing and unifying role. This is the case for the concepts of “sustainability” and “capacity development” [19–21]. After all, as per Judith Rodin (2014) and most other successive writers, resilience comes from integrating diverse concerns and strengths of a community. Turenne et al. comment on how presently, most writers do not share a consensus on the definitions, clarity, preconditions, or limits of the use of the term “health system resilience”. Turenne et al. see the hallmarks of a term that is not mature, not stable [13]. Biddle et al. also note that the term “resilience” is dynamic, complex, and in its infancy [7].

### Resilience for crises, for social reform, and for every day

Because of its elasticity, “resilience” has been pulled in multiple directions when it is used to guide thinking about health systems. There are three overlapping principal applications of resilience to health systems: 1**) Crisis resilience** refers to health system properties of high value during a crisis [13]; (2) **Social resilience also known as “transilience”** refers to health systems efforts to engage with broader social circles and wider environmental issues that affect health like differential access to power, knowledge, and resources [22] as well as community norms and social cohesion that can enable health and other systems to function and adapt in a crisis [23]. (3) **Everyday resilience** is the ability to handle the serious chronic challenges routinely facing health system managers [15, 24, 25]. There is still no consensus about whether these three types of resilience can or should focus on homeostasis—on restoring a system to an ideal baseline, or whether resilience refers to a healthy embrace of adaptation, transformation, and learning. Fridell et al. see a growing emphasis on a more adaptive understanding of resilience that embraces change and adapts to it [10].

To briefly summarize: the current consensus is that the concept of “resilience” refers to properties of health systems that are universally desired because they ease adaptation to change, but the specific properties and pathways to develop them are not fully agreed upon.

## Practical strategies to improve resilience

Defining agency over resilience, contextualizing it, and benchmarking it emerged as common themes. For a strategy to be practical, there has to be clarity over who is assigned what role and how they are to be accountable. Practicality demands that a feasible strategy be adapted to a particular context. For both implementation and evaluation, each strategy needs to have a system for making and using measurements.

### Who implements resilience strategies?

Health system resilience can be advanced or impeded by people inside and outside the health sector. A few papers saw resilience in broad whole of society terms that they called “community resilience” [23, 26]. The measures for community resilience addressed broad features of development, livelihoods, and social cohesion that were not bounded by the health system [23, 26], and hence difficult for health sector leaders to make practical.

Most papers focused heavily on strategies to be carried out by leaders from the government and noted the absolute need for top level support by national leadership. Recognizing the role of “whole of government” or “health in all policies”, Mckenzie et al. (2015) caution that entrenched interests outside the health sector can be quite challenging to change. Their case study of resilient responses to Ebola in Northern Nigeria looked specifically at management functions [27].

Managers at the sub-national or district level of the health system were repeatedly emphasized as being critical for resilient response to crises based on Uganda’s successful response to COVID-19 [12]. Mustafa et al’s (2022) review of 106 COVID 19 Response plans repeatedly flagged the need to strengthen sub-national capability for multi-sectoral collaboration to deliver services and keep community stakeholders coordinated in maintaining non-emergency services in a crisis [28]. Fridell et al’s scoping review also noted consensus around leadership with local governance based on a workforce with a mix of skills [10]. In the Ebola response in 2014, it was local level partnerships with community political leaders, NGOs, faith leaders and facility managers that executed the work of reaching citizens with effective messages about behavioral change that reduced transmission and identified chains of transmission [29, 30].

Implicit in identifying the role for managers at the district level was recognizing that mid-level managers participate in, but do not drive the policies that lead to health system change [11, 31]. National Public Health Institutes and Health Ministries were able in some cases to set up the structures that would activate sub-national, district public health officials to exercise their stewardship [5, 31].

### What should be done: the role of essential public health functions

The things that need doing for resilience will revolve around the tasks of sensing, deliberating, and acting. Decades of implementation work on “Essential Public Health Functions” (EPHFs) provide a ready-made to do list to increase sensing, deliberating, and acting [6, 31]. The idea of “essential public health functions” emerged in the late 1990s as a set of regional and national consensus-based lists of capabilities that national and sub-national public health departments had to carry out to create the physical and social conditions for large populations to be healthy. WHO convened an international Delphi panel to define a list of these essential functions [32]. National [33, 34] and regional [35, 36] initiatives followed to assess and improve the execution of these essential functions. Exercises to define and measure EPHFs have now been applied in over 100 countries [37]. Tools to define and assess EPHFs have been developed for Latin America, Western Pacific, Europe, Eastern Mediterranean regions as well as USA, Australia, UK, India, New Zealand, Israel, British Columbia [38], Mozambique, Botswana [39] and Angola [40]. Of note, WHO EMRO region developed and initially applied measurement of EPHFs in Qatar and Morocco [41]. An assessment tool for EMRO is now in the public domain [42]. EPHF measurement for a national health agency might be a process taking several months, but in a district can be accomplished in a few hours [39] using a combination of qualitative, quantitative, subjective, and objective responses by district health management teams.

There are variations in the details of EPHFs across regions, but all share a three-part structure with functions to support: (1) Sensing the current health, health threats, and health system assets; (2) Deliberation about what to do that is engaged with local stakeholders and aligned with local laws and culture; (3) Assurance that solutions are executed effectively. See Supplement 1 for a representative list of EPHFs from the WHO EMRO region that highlights the three-fold structure. The confluence of the EPHF construct with the resilience consensus is shown in Table 2. The WHO’s recognition that the EPHFs tools are a strategic pathway to resilience can leverage decades of progress in using EPHF tools to improve system performance [6, 31].

**Table 2:** All resilience frameworks share three broad steps of intelligence: sensing, deliberating, and acting. However, resilience can not be reduced to these three elements. Resilience emerges out of the purpose to which sensing, deliberating and acting are applied. Each cell characterizes known aspects of intelligence required for resilience

SOURCE	Afferent Sensing	Deliberation	Efferent Actions
[5]	“Awareness of capacities and risks”	Utilize lessons to transform	Mobilization and coordination of resources Self-regulation Adaptation Provide services in all contexts
[2].	Collect Knowledge	Integrate and analyze knowledge.	Build socially accepted legitimate institutions Manage interdependence
[10]	Learning, Absorption	Adaptation	Maintenance and transformation
[17] [16]	Aware of assets and threats Diverse connections	Integrate	Self-regulate Adapt

Applications of the EPHF tools to improve the resilience of health systems show contextual variability [38]. The absence of a universal consensus on what is included and excluded as an “essential” public health function reflects a recognition that context matters. The most common approach has been a national measurement of EPHFs leading to a national report or in some cases a regional set of national reports [36, 38]. Most of the time the evaluation cycle has stopped at evaluation without consequent intervention. Often the evaluation is restricted to national level omitting public health actors at the sub-national districts.

Our literature review found no evidence that there has ever been a prospective assessment of a national or district level project tying EPHF or resilience interventions to health system outputs or population health outcomes. The best explanation for the lack of field-level evaluations of EPHF improvement initiatives relates to the fragmented structure of health system financing where the bulk of funding goes to clinical services [27]. There is a window of hope that the COVID-19 pandemic has revealed the need for a better approach [6].

### How to implement practical strategies: the role of measurement

Kruk and co-authors were one of the earliest groups to conceptualize a resilience index composed of 25 elements that embody elements of the original Judith Rodin Rockefeller Foundation resilience formula (Aware/Diverse/Self-regulating/Integrated/Adaptive) [43]. A national adaptation of this index was prepared with the participation of national stakeholders from Bangladesh [18] and a second adaptation made for Pakistan was pilot-tested in 2021 [44].

The limitations of measurements have been noted. National measurements of compliance with International Health Regulations like the Joint External Evaluations have been faulted for their lack of follow up [37]. National measurements like the Global Health Security (GHS) Index which draws on these measurements have been found questionable after the USA scored extremely high on the GHS in 2019, but in practice had a disappointing performance in delivering health security during its COVID-19 epidemic [45].

For practical implementation that leads to change, measurements of resilience have to be integrated into a quality improvement cycle [46–48]. A pilot project in Botswana and Mozambique was able to develop stakeholder-endorsed measures of EPHFs suitable for the sub-national performance improvement cycles, but application and scale up of these cycles never occurred [39]. Measurement is not a panacea. Measurement divorced from a system of accountability to regularly revisit locally owned and generated EPHF-based assessments of resilience for public health performance improvement has repeatedly failed to trigger reform [38]. On the other hand, the complete absence of any evaluation of programs where resilience or EPHF measures were applied in a quality improvement cycle leaves a major gap in what is known about the impact of measurement [48]. A community-based trial of district level public health functions measurement to improve objective measures of resilience is a high priority for next steps. Other promising approaches that did not come up in our search strategy include social accountability that can be approached with community scorecards [49].

## Mechanisms for how actions alter resilience

People’s behaviors, performance, and trust-building are properties that adhere to all building blocks of a health system [50]. The interlinkage of health system components means governance improvements that alter health workforce capability and improve the information used in the system spillover to improve service delivery, finance, and medicine supply chains.

### Social features of resilience

The social aspects of resilience are well demonstrated in case studies like an analysis of Lebanon’s handling of the Syrian refugee crisis that was built on pre-existing social networks with diverse stakeholders [51]. Strong relationships between the public health officials and private health providers, school systems, faith communities, transport, law enforcement, agriculture etc. are a way to have off-budget surge capacity. A case study of Liberia’s Ebola response pointed out how pre-existing communications platforms with the community relied on treating community members as active participants and not passive recipients of health response efforts [52]. This facilitated Ebola response efforts and led to a fortuitous cycle of increased trust, improved communication and even more engagement. The Liberian case study stresses that the health system actors have to build public trust before a crisis. The Ebola response in Nigeria and Uganda also relied on previous social connections to partners [29].

This need to build social connections and networks before a crisis is the basis for synergy between everyday resilience and crisis resilience. One has to build a people-centered health system every day in order to have the social networks and trust that are critical for a resilient response to a crisis [24]. Gilson and co-authors’ list of resilience capacities stresses proactive efforts to build social capital by diffusing power and inclusion throughout an organization and outside it. To engage in this type of everyday resilience the organization will need to master skills in shared narrative and sense-making to align the diverse partners that have to work together [24]. Barasa et al. call these everyday resilience investments the “software” of resilience and note that this approach is inherently adaptive and uncommitted to preserving past structures in order to bounce back to them [15].

Every day investments in social connections for the health system build resilience, but so do every day investments in reaching groups who have been historically excluded or socially vulnerable. Haldane and Morgan (2020) point out that addressing the social and environmental concerns of marginalized groups is not only core to public health, but it pays a resilience dividend [22]. During a crisis reaching these groups with services and trusted messages becomes critical, because historically they have experienced, and in the future, they will continue to experience the highest losses in a crisis.

### Multisectoral linkages for resilience

Ensuring that the health sector is connected to other sectors of government has been termed, “health in all policies” [53]. Having functional relations among leaders of non-health government agencies (e.g., education, law enforcement, transportation, social services, environment, etc.) is not just an essential public health function [31], but also critical for resilience during a crisis [48]. Crisis response teams will draw on multiple government agencies and the teams will work better if they have worked together in other capacities for everyday tasks that they share for community well-being. There is a tendency for vertical “preparedness” planning to only see these multi-sectoral collaborations as useful in crisis response. But in fact, multisectoral work is basic to all of public health [31]. Another way that pre-crisis relations build linkage for resilience is their ability to assist with regional and global efforts in procurement of supplies and assistance during a crisis [54].

### The role of trust

Multiple papers emphasized that the health system has to build trust before a crisis [55, 17]. Crises will require collective coordinated activities by diverse elements of society that do not ordinarily collaborate [17]. The health system has a special role of being a bridge, but can only be such a bridge if it embraces this role and the call for repeated empathetic interaction with all members of a community [56]. Everyday resilience builds crisis resilience through inclusive provision of public health services with empathy and integrity to all members of a community [15, 51, 57].

## Sustaining resilience improvements by defragmentation

Fragmentation of health system financing and organization was cited as the key barrier to resilience. The funding streams in both high income and low income health systems come from multiple levels of government and multiple programs [58]. Public health agencies at national and sub-national level face verticalized sequestered budgets with funds that are earmarked to specific diseases, treatment programs or technologies. Campbell and co-authors who shared a particular interest in Perinatal Maternal, Newborn, and Child health (PMNCH) recognized that fragmentation was blocking their own particular agenda and called for integration of their vertical efforts into primary health care [59]. Ordinary people want clinics that can take care of their whole body and their whole family, but fragmentation induces care options that focus on single problems or sub-populations. PMNCH could reach more people if it could be part of a person-centered system rather than a problem-centered one.

Fragmentation’s root cause stems from the practice of budgeting and organizing health systems around separate strategic objectives. As Ramalingam notes, top-down, blueprint planning approaches have been integral to government and business since the early 20th century [60]. The units that make up the building blocks of a health system are given their objectives and task lists independently. The hard work of connecting and integrating the units to each other or to make them able to sense and respond to emerging problems together is seldom a pre-specified top-down task. Defragmentation does not call for abandonment of the top-down approach, but the augmentation of these multiple units by forging linkages across fragmented programs. The workforce that can do this linking would naturally be part of governmental public health departments. The tools they need would be the ability to observe the assets present in the system as well as their relevance to emerging health problems. The integrative work of coordinating multiple siloed projects in a system is inherent in efforts to improve capacity in essential public health functions. The EPHF make it essential to deliberate together based on data on assets and problems about how to respond to emerging health problems. Hence adding EPHF capability to all members of the public health workforce can bring coherence to a siloed and fragmented system.

## Summary and Conclusion

Improving resilience will not look like a standard implementable project with a sequence of step 1, step 2, and step 3. Resilience emerges from doing many things to nudge a system towards the sentient action state outlined in Table 2. Lack of resilience is a systems problem rooted in fragmentation whose origin was management by strategic objectives. The cure for narrow, segmented strategies is broad cross-cutting strategies. Yes, one can implement practical *strategies* to counteract an emergent systemic weakness brought on by too much strategic focus. Ultimately, the pathway to resilience must include integrative sensing, deliberating, and doing throughout a health system.

- Assign a unit of the health system that will be explicitly responsible for resilience, specified as bringing out sensing, deliberating, and doing as a system-wide responsibility. Context will decide, but in most cases the national and sub-national public health workforce will need to lead resilience building efforts [24, 30]. Public health institutes at national and sub-national level are an obvious choice [5, 31]. In applications of the viable system model community stakeholders are engaged for advice on how a local system can be adapted [61].
- Set up policies and legislation that assign the resilience responsibility to national and sub-national teams [62]. There is a danger that this will be mis-interpreted as one more fragmented unit, i.e. “a resilience squad” or a “public health preparedness office’. However, noting the homology of resilience to the more frequently operationalized efforts to improve essential public health functions throughout a health system can defend against this pitfall.
- Convene broad stakeholders in a conversation about context-relevant resilience indicators [56, 63]. Drawing on EPHF checklists will accelerate this effort [42]. Resilience indicators coming from EPHF checklists would naturally show their relevance to everyday concerns in service delivery for vertical programs and vulnerable groups [14, 18].
- Embed annual or quarterly cycles of measurement of contextualized resilience indicators into a total quality management cycle where the resilience scores drive local performance improvement plans that can be costed and met with financing [39].
- Assist with technical support in the areas of social science, inclusion, quality improvement, coaching and workforce development [12, 30].
- Accompany resilience improvement with evaluation to assess impact and lead to modification and learning in the effort [64].
- Invest in lateral learning [65]. By benchmarking performance, one can learn which sub-national units are doing well in resilience improvement, and they can become catalysts for change in units that are slightly lagging.

The review identified zero empirical prospective field trials that have showed longitudinal changes in a quantitative or qualitative assessment of resilience. The chief obstacle to implementing resilience interventions that emerged was fragmented silo-based organization of the work of many public health systems. When health systems become organized around specific-diseases and sub-populations they and those who govern them use specific key service performance indicators. Since these indicators rarely call on a worker to help their workplace or community become aware of emerging health threats or to cultivate connections to community resources that could help respond in a crisis, these key resilience capabilities and EPHFs stay out of sight and off the agenda.

Our review revealed many practical opportunities that can put resilience into practice. The elements of resilience (sensing, deliberating, doing) look exactly the same as EPHFs. The extensive toolkit of EPHF measures for accountability can accelerate local contextualization of benchmarks for resilience. Importantly these resilience and EPHF benchmarking measures can be applied longitudinally and reacted to with resourced improvement plans. Although benchmarking and improvement plans are tools of health system governance their effect spills over to affect all components (e.g. building blocks) of the health system.

There is enough known right now to support pilot interventions that could be implemented to improve resilience. Future work in this area needs to capitalize on the open window of the current high prioritization of resilience at the highest level [6]. The next big step in resilience will be field implementations of resilience projects that are prospectively evaluated with mixed methods.

### Electronic supplementary material

Below is the link to the electronic supplementary material.

Supplementary Material 1

Supplementary Material 2

Supplementary Material 3

## Data Availability

All papers reviewed in the systematic review are listed in the bibliography and can be accessed publicly.

## Abbreviations

EMRO: Regional Office for the Eastern Mediterranean (of the WHO)
EPHF: Essential public health functions
EPR: Emergency preparedness and response
GHS: Global health security
HEDRM: health emergency and disaster risk management
HEW: Health extension worker
MeSH: Medical subject heading
NCD: Non communicable disease
NPHI: National public health institute
PHC: Primary health care
PMNCH: Prenatal maternal neonatal child health
UNICEF: United Nations Children’s Fund
WHO: World Health Organization
