# Supplementary material for: Practical strategies to achieve resilient health systems: results from a scoping review
Source: BMC Health Serv Res. 2024 Mar 6;24:297. doi: 10.1186/s12913-024-10650-8 (PMC10918906; doi:10.1186/s12913-024-10650-8)
Supplement: Supplementary file 1 — Supplementary Material 1 [file 12913_2024_10650_MOESM1_ESM.docx]

# Practical Strategies to Achieve Resilient Health Systems

# Results from a Scoping Review

**Supplement 1**.

Example of an Essential Public Health Functions (EPHF) list [1]. This example from the Eastern Mediterranean Regional Office (EMRO) of the WHO. The table maps how each function contributes to resilient intelligence in column 1. EMRO’s original documented classified each function as core or enabling.

| **Resilient Intelligence Task** | **Public Health Functions** | **Core or Enabling** |
| --- | --- | --- |
| Afferent sensing | Surveillance and monitoring of health determinants, risks, morbidity and mortality | Core |
| Deliberation | Effective health governance, public health legislation, financing and institutional structures | Enabling |
| Deliberation | Public health research to inform and influence policy and practice | Enabling |
| Efferent action | Sufficient and competent workforce for effective public health delivery | Enabling |
| Efferent action | Health protection including management of environmental, food, toxicological and occupational safety | Core |
| Efferent action | Health promotion and disease prevention through population and personalized interventions, including action to address social determinants and health inequity | Core |
| Efferent action | Preparedness and public health response to disease outbreaks, natural disasters and other emergencies | Core |
| Efferent action | Communication and social mobilization for health | Enabling |

References

1. World Health Organization: **Assessment of essential public health functions in countries of the Eastern Mediterranean Region: assessment tool**. 2017.
