# Supplementary material for: Practical strategies to achieve resilient health systems: results from a scoping review
Source: BMC Health Serv Res. 2024 Mar 6;24:297. doi: 10.1186/s12913-024-10650-8 (PMC10918906; doi:10.1186/s12913-024-10650-8)
Supplement: Supplementary file 2 — Supplementary Material 2 [file 12913_2024_10650_MOESM2_ESM.docx]

**Practical Strategies to Achieve Resilient Health Systems Results from a Scoping Review**

**Supplement 2 Alignment of Practical Strategies with Identified Priorities towards building resilient health systems.**

The Eastern Mediterranean Regional Office of the WHO identified a comprehensive list of priorities in building resilient health systems [1]. This supplement shows how the practical strategies identified in this review correspond to many of these priorities. Embedded at the “heart” of the WHO EMRO resilience priorities is the core practical resilience strategy of benchmarking, gap-finding, and resourcing strategic essential public health functions that support resilience.

| **WHO EMRO Resilience Priorities** | **WHO EMRO Sub-priorities** | **Resilience Practical Strategies** |
| --- | --- | --- |
| 1. Strengthen health emergency and disaster risk management (HEDRM) | - Use reviews to identify gaps in all HS components - Strengthen community engagement to prevent detect and respond | - Customizing EPHF indicators to context allows them to reflect gaps in HEDRM functions - EPHF already call for community engagement |
| 2. Building institutions for public health | - Establish a multisectoral platform to convene stakeholders from health and other sectors - Build institutional and individual capacities to cultivate a “learning health system” - Transparent decision-making | - Convening and engaging multisectoral stakeholders is a typical EPHF - Institutionalizing public health system sentience and learning is an EPHF - Building trust through transparent decision making is an EPHF |
| 1. Build PHC oriented models of care | - Invest in continuous quality improvement - Link community health approach to facility-based services | - Linking both population level and individual health perspective is part of EPHF - Using local research to improve the public health system is an EPHF |
| 1. Enhance and scale up a fit-for-purpose and fit-to-practice health workforce | - Identify the competencies required to deliver EPHFs - Benchmark EPHFs and map all personnel to EPHFs for gap assessments to inform the development and strengthening of the workforce. | - Developing and maintaining a competent public health workforce is an EPHF - Benchmarking EPHFs is a practical strategy for resilience identified in this paper |
| 1. Promote equity and financial protection | - Make the economic case for EPHFs to ministries of finance - Use health accounts to track progress towards UHS and health security | - The performance improvement plans that follow EPHF gap identification must be costed to be actionable - Tracking PH spending on core PH capabilities is critical to see the gaps in this area |
| 1. Promote access to medicines | - Revamp supply chain systems | - Assuring functional supply chains is an EPHF |
| 1. Integrate policy and planning for resilience | - Joint planning of stakeholders within health and allied sectors - Implement joint actions | - Convening multi-sectoral stakeholders and mobilizing them is an essential public health function. |

1. WHO Regional Committee for the Eastern Mediterranean: **Building resilient health systems to advance universal health coverage and ensure health security in the Eastern Mediterranean Region**. In: *Sixty Ninth Session.* vol. EM/RC69/4. Cairo: WHO; 2022.
