# Supplementary material for: Practical strategies to achieve resilient health systems: results from a scoping review
Source: BMC Health Serv Res. 2024 Mar 6;24:297. doi: 10.1186/s12913-024-10650-8 (PMC10918906; doi:10.1186/s12913-024-10650-8)
Supplement: Supplementary file 3 — Supplementary Material 3 [file 12913_2024_10650_MOESM3_ESM.docx]

# Practical Strategies to Achieve Resilient Health Systems Results from a Scoping Review

| **Supplement 3 Short summaries of 65 Extracted Papers Sorted by Primary Code** | | |
| --- | --- | --- |
| **Primary code** | **Short summary** | **Reference** |
| Defines Resilience | There is too much focus on shock absorption. Wider framing should adapt to local context, recognize complexity, consider cumulative stresses like NCDs and costs. | [1] |
| Defines Resilience | Distinguish EPR (emergency preparedness and response) and inherent system resilience (ISR) | [2] |
| Defines Resilience | Resilience requires: Crisis sensing, Opportunity seizing, Reconfiguration  Requires cooperation between academic and executive bodies. Requires public participation in health programs for social trust. | [3] |
| EPHF | Needs-based training is not a solution for everything. What happens after training and when the worker gets to the organization matters too. | [4] |
| EPHF | Singapore's COVID-19 playbook emphasized: planning, good communication, execute containment measures, maintain services, access crisis financing, legal foundation for all of the above. | [5] |
| EPHF | Must: 1. Strengthen EPHFs, 2. Collaborate internationally, 3. Address inequalities, | [6] |
| EPHF | Health workforce competencies should include coping with uncertainties, transformative leadership and communication, emergency risk communication, empathy, commitment, dedication and resilience. | [7] |
| EPHF | Introducing formal education programs into health care systems and health professional training courses to address the acquisition of responsiveness and resilience knowledge and skills. | [8] |
| EPHF | Set up a high-level multisectoral mechanism to oversee health system resilience building, with clear roles and responsibilities for its stakeholders Benchmark the functions, sub-functions and tasks in the delivery of EPHFs, map all personnel engaged and conduct competency- gap assessments to inform the development and strengthening of workforce, with a focus on public health capacity and emergency preparedness and response competencies WHO deliverable is to identify the competencies required to deliver EPHFs. WHO also plans to make a business case for EPHFs in region | [9] |
| EPHF | Appoint and institutionalize a steward for EPHFs, e.g. the National Public Health Institutes (NPHIs). "Systematic strengthening of the EPHFs inherently builds resilience in health systems." | [10] |
| EPHF | Three components matter: Multisectoral policy and action, Empowered people and communities, Integrated health services emphasis on Primary Care and EPHF. | [11] |
| Everyday Resilience | COVID- 19 has shown that health systems are resilient when they are already strong. Leave aside a narrow crisis management approach and seek to better understand the  complexity of the dynamics and interactions involved in developing everyday strength. | [12] |
| Everyday Resilience | Rwandan example was to build improved supply chain, data for decisions, improved communications during years prior to pandemic and call on these capabilities during the pandemic | [13] |
| Everyday Resilience | Adaptive resilience and the ability to adjust and be flexible may be more important than planned resilience (preparedness) when the crisis comes, especially in countries where resources are scarce and it is therefore difficult to prepare for everything | [14] |
| Everyday Resilience | Nurture the power of leaders to re-frame challenges, encourage mindful staff engagement, develop social networks, within and outside organizations. | [15] |
| Everyday Resilience | Micro-governance interventions and improvement of mid-level leadership practice can be transformative. Resilience is a process involving many strategies deployed in combination. | [16] |
| Everyday Resilience | Calling on countries to invest more in resilience implies the importance of good plans on what to actually do about resilience. | [17] |
| Everyday Resilience | Note that " existing health systems capacities and political leadership determined how responses unfolded, while emergency plans or pandemic preparedness documents were not fit-for-purpose" | [18] |
| Everyday Resilience | Everyday resilience is enhanced by managers who have communication skills, emotional competence, and supportive oversight. | [19] |
| Everyday Resilience | Resilience entails ensuring long term stability of resources (financial, physical, human, information), responding efficiently with the available resources and strengthening governance to formulate long term health strategy, ensure accountability, transparency and stakeholder involvement, as well as use evidence for monitoring and performance evaluation. | [20] |
| Fragmentation | Calls out fragmentation as an issue for the Perinatal Maternal Neonatal Child Health (PMNCH) community and asks for integration of vertical concerns like PMNCH into PHC. | [21] |
| Fragmentation | Fragmentation is the most significant problem. Accountability becomes weak. Community becomes confidence low. Vertical programs have unintended consequences. | [22] |
| Measuring Resilience | Offers a list of 20 indicators at level of community. Not specified who will apply them. | [23] |
| Measuring Resilience | "[There is a] mismatch between the conceptual models of health system resilience and the way resilience is understood and applied in empirical research both in terms of the breadth of health system factors considered and in terms of the resilience dimensions which are taken into account" | [24] |
| Measuring Resilience | Different health systems seemed to have little influence on the specific combinations of capacities of the health workforce. This challenges our understanding of health systems and calls for a reconceptualisation of the institutional prerequisites of health system resilience, in order to fully grasp the manifold and unique health workforce contribution. | [25] |
| Measuring Resilience | Review sought to identify metrics that relate to the handling of shocks on health systems. There are not a lot of governance metrics in the literature yet. | [26] |
| Measuring Resilience | Resilience is context dependent iterative need advance assessments of capacities, weaknesses. Invest before crisis | [27] |
| Measuring Resilience | Stakeholder convening can develop local contextualized definitions of implementable resilience indicators to be used as a checklist for accountability. Consensus is possible | [28] |
| Measuring Resilience | An implementation-oriented health system resilience framework could help translate the important components of a health system identified in this review into specific capacities that actors in the health system could work to develop to improve resilience to public health crises. | [29] |
| Measuring Resilience | Practical tool kits can be developed in 5 phases:  Phase 0 adapt toolkit to context.  Phase 1 qualitative assessment of baseline via facilitated group discussion of "normal" and "what if" scenarios  Phase 2 Quantify data on indicators about current normal function and simulate changes under "what if"  Phase 3 Summarize  Phase 4A Share results with stakeholders  Phase 4B Action planning for implementable improvement (inclusive process) | [30] |
| Multisectorality | Systems thinking approach, clear policies and guidelines, collaboration between various actors, ministries, & departments, effective communication, and country specific action plan formulating short and long term strategies. | [31] |
| Multisectorality | Six elements required, adequate workforce, supplies, health information systems, facilities, financing, strong public sector | [32] |
| Multisectorality | Eighteen African countries gathered to share best practices in improving PHC. | [33] |
| Multisectorality | Need greater participation of stakeholders to maintain non-emergency services in a crisis. | [34] |
| PHC | Collaboration with stakeholders, diversification of the health system, enough infrastructure and human resources for health, a thorough communicable disease response, and integration of refugees into the health system are all priorities. | [35] |
| PHC | Declines in all 10 countries in health care utilization. Some was attributed to lower health care needs, some they say is "failure of health system resilience" | [36] |
| PHC | Rather than putting a brake on major reform, the Irish government utilised the shock of COVID-19 to progress significantly its flagship health reform programme, Slaintecare, to advance universal healthcare. | [37] |
| PHC | Resilience requires strong PHC, surge capacity, surveillance and health info systems, Domestic production of med tech, Governance, Human resource capacity | [38] |
| PHC | Primary level of a three layer system includes essential public health functions. Important to work at pre-crisis phase. | [39] |
| Social inputs to resilience | Collateral pathways is a form of redundancy offering extra components that can be called on later. Build this strength by partnering as part of everyday system operations. | [40] |
| Social inputs to resilience | Local health systems had 3 phases: Prepare, Adapt, Learn | [41] |
| Social inputs to resilience | Building and maintaining public trust through community engagement and participation is key: trust in governments, public services and health systems represents social capital built up over time through active two-way communication and engagement with populations, communities and civil society. | [42] |
| Supplies | Historically Tamil Nadu had had an ongoing relationship with Tamil Nadu Medical Services Corporation Limited going back to 1995 and this private company helped with logistics and supply chain. This helped during crisis. | [43] |
| To Do/Gaps | "There is a gap in available practical technical resources on how countries can develop legislative instruments, policies and plans to enable an integrated approach” | [44] |
| Trust | Can use community connections to increase access to care and to increase the advocacy efforts | [45] |
| Trust | Factors for resilience in Liberian Ebola crisis : strong leadership, tight bonds and sense of kinship at the community level; trusted communication channels; and trust among various health system stakeholders These factors operated mainly by facilitating collective actions within communities, and effective dissemination of response initiatives from other levels of the health system | [46] |
| Trust | The importance of public trust and community engagement as a base to build resilience and this is through information provision, consultation, participation, partnerships, and double channel community empowerment by giving them trusted information and taking their feedback and thoughts. communities are partners not recipients so we need to build platforms for them to ensure they receive the health service properly. | [47] |
| Trust | Building trust entails longstanding collaboration, a cadre of trained staff and a shared vision from a collective sense of ownership. | [48] |
| Trust | Health policies for resilience need to reform anti-feminist power structures. Must keep Gender, Equity and Justice in focus. | [49] |
| Trust | Focus on five elements: Access to health care, Social connectedness, Organizational resilience  Psychological resilience, Needs of at risk individuals | [50] |
| Who does what | Identify bench strength of health workforce and upskill them | [51] |
| Who does what | Health extension workers (HEWs) and Health Development Army can be a tool for resilience because they links health system to every household. | [52] |
| Who does what | To address the insecurity of healthcare employees, giving incentives, motives, and staff commitment will help staff be retained and supported. | [53] |
| Who does what | Opportunities for improvement include correcting the absence of dedicated public health professionals resulting in severe compromises in routine primary health programs. | [54] |
| Who does what | District level integration led to resilience under Covid for Uganda. Best practice is to have teams capable of investigating and responding. Support the teams with structures that sustain and advance technical capability. Empower district managers to integrate all workers and create transparency in workloads. | [55] |
| Who does what | The key determinant of the health system strengthening emphasized in this paper is well trained healthcare workers | [56] |
| Who does what | Community engagement plays a role in resilience, and community health workers serve as a bridge between communities and health system | [57] |
| Who does what | Paper focuses on "central role of improving community ownership towards effective outbreak response" | [58] |
| Who does what | Roles for governance, planning, partnering, trust-building, risk analysis, surveillance, drills, human resources, financing, communication, learning | [59] |
| Who does what | Taiwan and Singapore had SARS 2003. Korea had MERS. They reformed their PH agencies and developed info systems because of learning after these crises. | [60] |
| Who does what | County respondents prioritized "self-regulating" and "diverse" as resilience elements more than global respondents. Global actors prioritized "integrated" as a key aspect of resilience. | [61] |
| Who does what | Focus on human resources is important | [62] |
| Who does what | Resilience requires 1) organized community groups linked to the health system, 2) an effective community health workforce within strong health systems, 3) adaptable human resource structures and service delivery models, 4) training and preparedness, and 5) strong government leadership with decentralized decision making. | [63] |
| Who does what | Notes that resilience involves (1) working multisectorally/intersectorally, (2) moving from fragmentation to integration, (3) ensuring implementation and knowledge exchange, and (4) rethinking resilience and embracing antifragility | [64] |
| Who does what | Structured Operational Research and Training IniTiative called “SORT-IT" trained 895 health workers. Surveyed during COVID. Result: past trainees were applying this training to fight COVID. | [65] |

1. Haldane V, De Foo C, Abdalla SM, Jung AS, Tan M, Wu S, Chua A, Verma M, Shrestha P, Singh S *et al*: **Health systems resilience in managing the COVID-19 pandemic: lessons from 28 countries**. *Nature medicine* 2021, **27**(6):964-980.

2. Karamagi HC, Titi-Ofei R, Kipruto HK, Seydi AB, Droti B, Talisuna A, Tsofa B, Saikat S, Schmets G, Barasa E *et al*: **On the resilience of health systems: A methodological exploration across countries in the WHO African Region**. *PloS one* 2022, **17**(2):e0261904.

3. Pilevari N, Shiva MV: **Country-Wide Resilience Model for the Health System: A Case Study on Iran, under Coronavirus Outbreak**. *Iranian journal of public health* 2021, **50**(4):806-815.

4. Board on Health Sciences P, Institute of M, National Academies of Sciences E, Medicine. In: *Global Health Risk Framework: Resilient and Sustainable Health Systems to Respond to Global Infectious Disease Outbreaks: Workshop Summary.* edn. Washington (DC): National Academies Press (US)

Copyright 2016 by the National Academy of Sciences. All rights reserved.; 2016.

5. Chua AQ, Tan MMJ, Verma M, Han EKL, Hsu LY, Cook AR, Teo YY, Lee VJ, Legido-Quigley H: **Health system resilience in managing the COVID-19 pandemic: lessons from Singapore**. *BMJ global health* 2020, **5**(9).

6. Collins T, Akselrod S, Bloomfield A, Gamkrelidze A, Jakab Z, Placella E: **Rethinking the COVID-19 Pandemic: Back to Public Health**. *Annals of global health* 2020, **86**(1):133.

7. Czabanowska K, Kuhlmann E: **Public health competences through the lens of the COVID-19 pandemic: what matters for health workforce preparedness for global health emergencies**. *Int J Health Plann Manage* 2021, **36**(S1):14-19.

8. Kyoto MA: **Meeting Emerging Challenges: Toward Responsive and Resilient Health Systems**. In*.* Edited by Alliance M; 2015.

9. WHO Regional Committee for the Eastern Mediterranean: **Building resilient health systems to advance universal health coverage and ensure health security in the Eastern Mediterranean Region**. In: *Sixty Ninth Session.* vol. EM/RC69/4. Cairo: WHO; 2022.

10. World Health Organization: **21st century health challenges: can the essential public health functions make a difference?: discussion paper**. 2021.

11. World Health Organization and United Nations Children's Fund (UNICEF): **Operational framework for primary health care: transforming vision into action**. 2020.

12. Alami H, Lehoux P, Fleet R, Fortin JP, Liu J, Attieh R, Cadeddu SBM, Abdoulaye Samri M, Savoldelli M, Ag Ahmed MA: **How Can Health Systems Better Prepare for the Next Pandemic? Lessons Learned From the Management of COVID-19 in Quebec (Canada)**. *Frontiers in public health* 2021, **9**:671833.

13. Binagwaho A, Hirwe D, Mathewos K: **Health System Resilience: Withstanding Shocks and Maintaining Progress**. *Global health, science and practice* 2022, **10**(Suppl 1).

14. Forsgren L, Tediosi F, Blanchet K, Saulnier DD: **Health systems resilience in practice: a scoping review to identify strategies for building resilience**. *BMC health services research* 2022, **22**(1):1173.

15. Gilson L, Barasa E, Nxumalo N, Cleary S, Goudge J, Molyneux S, Tsofa B, Lehmann U: **Everyday resilience in district health systems: emerging insights from the front lines in Kenya and South Africa**. *BMJ global health* 2017, **2**(2):e000224.

16. Gilson L, Ellokor S, Lehmann U, Brady L: **Organizational change and everyday health system resilience: Lessons from Cape Town, South Africa**. *Social science & medicine (1982)* 2020, **266**:113407.

17. Kluge H, Martín-Moreno JM, Emiroglu N, Rodier G, Kelley E, Vujnovic M, Permanand G: **Strengthening global health security by embedding the International Health Regulations requirements into national health systems**. *BMJ global health* 2018, **3**(Suppl 1):e000656.

18. Neill R, Neel AH, Cardona C, Bishai D, Gupta S, Mohan D, Jain N, Basu S, Closser S: **Everyday capabilities were a path to resilience during COVID-19: A case study of five countries**. *Health policy and planning* 2022.

19. Nzinga J, Boga M, Kagwanja N, Waithaka D, Barasa E, Tsofa B, Gilson L, Molyneux S: **An innovative leadership development initiative to support building everyday resilience in health systems**. *Health policy and planning* 2021, **36**(7):1023-1035.

20. Thomas S, Sagan A, Larkin J, Cylus J, Figueras J, Karanikolos M: **Strengthening health systems resilience: key concepts and strategies**. 2020.

21. Campbell J, Cometto G, Rasanathan K, Kelley E, Syed S, Zurn P, de Bernis L, Matthews Z, Benton D, Frank O *et al*: **Improving the resilience and workforce of health systems for women's, children's, and adolescents' health**. *BMJ (Clinical research ed)* 2015, **351**:h4148.

22. McKenzie A, Abdulwahab A, Sokpo E, Mecaskey JW: **Creating the Foundation for Health System Resilience in Northern Nigeria**. *Health systems and reform* 2016, **2**(4):357-366.

23. Bhandari S, Alonge O: **Measuring the resilience of health systems in low- and middle-income countries: a focus on community resilience**. *Health research policy and systems* 2020, **18**(1):81.

24. Biddle L, Wahedi K, Bozorgmehr K: **Health system resilience: a literature review of empirical research**. *Health policy and planning* 2020, **35**(8):1084-1109.

25. Burau V, Falkenbach M, Neri S, Peckham S, Wallenburg I, Kuhlmann E: **Health system resilience and health workforce capacities: Comparing health system responses during the COVID-19 pandemic in six European countries**. *Int J Health Plann Manage* 2022, **37**(4):2032-2048.

26. Fleming P, O'Donoghue C, Almirall-Sanchez A, Mockler D, Keegan C, Cylus J, Sagan A, Thomas S: **Metrics and indicators used to assess health system resilience in response to shocks to health systems in high income countries-A systematic review**. *Health policy (Amsterdam, Netherlands)* 2022.

27. Kruk ME, Ling EJ, Bitton A, Cammett M, Cavanaugh K, Chopra M, El-Jardali F, Macauley RJ, Muraguri MK, Konuma S *et al*: **Building resilient health systems: a proposal for a resilience index**. *BMJ (Clinical research ed)* 2017, **357**:j2323.

28. Meyer D, Bishai D, Ravi SJ, Rashid H, Mahmood SS, Toner E, Nuzzo JB: **A checklist to improve health system resilience to infectious disease outbreaks and natural hazards**. *BMJ global health* 2020, **5**(8).

29. Nuzzo JB, Meyer D, Snyder M, Ravi SJ, Lapascu A, Souleles J, Andrada CI, Bishai D: **What makes health systems resilient against infectious disease outbreaks and natural hazards? Results from a scoping review**. *BMC public health* 2019, **19**(1):1310.

30. Rogers HL, Barros PP, Maeseneer J, Lehtonen L, Lionis C, McKee M, Siciliani L, Stahl D, Zaletel J, Kringos D: **Resilience Testing of Health Systems: How Can It Be Done?** *International journal of environmental research and public health* 2021, **18**(9).

31. Balqis-Ali NZ, Fun WH, Ismail M, Ng RJ, Jaaffar FSA, Low LL: **Addressing Gaps for Health Systems Strengthening: A Public Perspective on Health Systems' Response towards COVID-19**. *International journal of environmental research and public health* 2021, **18**(17).

32. Kamal-Yanni M: **Never Again: Building resilient health systems and learning from the Ebola crisis**: Oxfam International; 2015.

33. Karamagi H, Titi-Ofei R, Amri M, Zombre S, Kipruto H, Seydi AB, Avortri G, Nabyonga J, Tumusiime P: **Cross country lessons sharing on practices, challenges and innovation in primary health care revitalization and universal health coverage implementation among 18 countries in the WHO African Region**. *The Pan African medical journal* 2022, **41**:159.

34. Mustafa S, Zhang Y, Zibwowa Z, Seifeldin R, Ako-Egbe L, McDarby G, Kelley E, Saikat S: **COVID-19 Preparedness and Response Plans from 106 countries: a review from a health systems resilience perspective**. *Health policy and planning* 2022, **37**(2):255-268.

35. Ammar W, Kdouh O, Hammoud R, Hamadeh R, Harb H, Ammar Z, Atun R, Christiani D, Zalloua PA: **Health system resilience: Lebanon and the Syrian refugee crisis**. *Journal of global health* 2016, **6**(2).

36. Arsenault C, Gage A, Kim MK, Kapoor NR, Akweongo P, Amponsah F, Aryal A, Asai D, Awoonor-Williams JK, Ayele W *et al*: **COVID-19 and resilience of healthcare systems in ten countries**. *Nature medicine* 2022, **28**(6):1314-1324.

37. Burke S, Parker S, Fleming P, Barry S, Thomas S: **Building health system resilience through policy development in response to COVID-19 in Ireland: From shock to reform**. *The Lancet regional health Europe* 2021, **9**:100223.

38. Sundararaman T, Muraleedharan VR, Ranjan A: **Pandemic resilience and health systems preparedness: lessons from COVID-19 for the twenty-first century**. *Journal of social and economic development* 2021, **23**(Suppl 2):1-11.

39. Zhao F, Bali S, Kovacevic R, Weintraub J: **A three-layer system to win the war against COVID-19 and invest in health systems of the future**. *BMJ global health* 2021, **6**(12).

40. Barasa E, Mbau R, Gilson L: **What Is Resilience and How Can It Be Nurtured? A Systematic Review of Empirical Literature on Organizational Resilience**. *International journal of health policy and management* 2018, **7**(6):491-503.

41. Juárez-Ramírez C, Reyes-Morales H, Gutiérrez-Alba G, Reartes-Peñafiel DL, Flores-Hernández S, Muños-Hernández JA, Escalante-Castañón A, Malo M: **Local Health Systems Resilience in Managing the COVID-19 Pandemic: Lessons from Mexico**. *Health policy and planning* 2022.

42. WHO: **Building health systems resilience for universal health coverage and health security during the COVID-19 pandemic and beyond**, vol. (WHO/UHL/PHC-SP/2021.01: World Health Organization; 2021.

43. Adithyan GS, Sundararaman T: **Good public health logistics for resilient health systems during the pandemic: Lessons from Tamil Nadu**. *Indian journal of medical ethics* 2021, **Vi**(3):1-10.

44. World Health Organization: **Health systems resilience toolkit: a WHO global public health good to support building and strengthening of sustainable health systems resilience in countries with various contexts**. 2022.

45. Alameddine M, Fouad FM, Diaconu K, Jamal Z, Lough G, Witter S, Ager A: **Resilience capacities of health systems: Accommodating the needs of Palestinian refugees from Syria**. *Social science & medicine (1982)* 2019, **220**:22-30.

46. Alonge O, Sonkarlay S, Gwaikolo W, Fahim C, Cooper JL, Peters DH: **Understanding the role of community resilience in addressing the Ebola virus disease epidemic in Liberia: a qualitative study (community resilience in Liberia)**. *Global health action* 2019, **12**(1):1662682.

47. Barker KM, Ling EJ, Fallah M, VanDeBogert B, Kodl Y, Macauley RJ, Viswanath K, Kruk ME: **Community engagement for health system resilience: evidence from Liberia's Ebola epidemic**. *Health policy and planning* 2020, **35**(4):416-423.

48. Grimm PY, Wyss K: **What makes health systems resilient? A qualitative analysis of the perspectives of Swiss NGOs**. *Globalization and health* 2022, **18**(1):55.

49. Mansour W, Arjyal A, Hughes C, Gbaoh ET, Fouad FM, Wurie H, Kyaw HK, Tartaggia J, Hawkins K, Than KK *et al*: **Health systems resilience in fragile and shock-prone settings through the prism of gender equity and justice: implications for research, policy and practice**. *Conflict and health* 2022, **16**(1):7.

50. Wulff K, Donato D, Lurie N: **What is health resilience and how can we build it?** *Annual review of public health* 2015, **36**:361-374.

51. Grimm PY, Oliver S, Merten S, Han WW, Wyss K: **Enhancing the Understanding of Resilience in Health Systems of Low- and Middle-Income Countries: A Qualitative Evidence Synthesis**. *International journal of health policy and management* 2021.

52. Admasu KB: **Designing a Resilient National Health System in Ethiopia: The Role of Leadership**. *Health systems and reform* 2016, **2**(3):182-186.

53. Ager AK, Lembani M, Mohammed A, Mohammed Ashir G, Abdulwahab A, de Pinho H, Delobelle P, Zarowsky C: **Health service resilience in Yobe state, Nigeria in the context of the Boko Haram insurgency: a systems dynamics analysis using group model building**. *Conflict and health* 2015, **9**:30.

54. Al Khalili S, Al Maani A, Al Wahaibi A, Al Yaquobi F, Al-Jardani A, Al Harthi K, Alqayoudhi A, Al Manji A, Al Rawahi B, Al-Abri S: **Challenges and Opportunities for Public Health Service in Oman From the COVID-19 Pandemic: Learning Lessons for a Better Future**. *Frontiers in public health* 2021, **9**:770946.

55. Alilio M, Hariharan N, Lugten E, Garrison K, Bright R, Owembabazi W, Inyang U, Hassan SE, Saldana K: **Strategies to Promote Health System Strengthening and Global Health Security at the Subnational Level in a World Changed by COVID-19**. *Global health, science and practice* 2022, **10**(2).

56. Ayanore MA, Amuna N, Aviisah M, Awolu A, Kipo-Sunyehzi DD, Mogre V, Ofori-Asenso R, Gmanyami JM, Kugbey N, Gyapong M: **Towards Resilient Health Systems in Sub-Saharan Africa: A Systematic Review of the English Language Literature on Health Workforce, Surveillance, and Health Governance Issues for Health Systems Strengthening**. *Annals of global health* 2019, **85**(1).

57. Gebremeskel AT, Otu A, Abimbola S, Yaya S: **Building resilient health systems in Africa beyond the COVID-19 pandemic response**. *BMJ global health* 2021, **6**(6).

58. Khalil M, Mataria A, Ravaghi H: **Building resilient hospitals in the Eastern Mediterranean Region: lessons from the COVID-19 pandemic**. *BMJ global health* 2022, **7**(Suppl 3).

59. Khan Y, O'Sullivan T, Brown A, Tracey S, Gibson J, Généreux M, Henry B, Schwartz B: **Public health emergency preparedness: a framework to promote resilience**. *BMC public health* 2018, **18**(1):1344.

60. Kim J, Moon J, Jung TY, Kim W, Yoo HC: **Why Have the Republic of Korea, Taiwan, and Singapore Coped Well with COVID-19 and What Are the Lessons Learned from Their Experiences?** *Yonsei medical journal* 2022, **63**(3):296-303.

61. Ling EJ, Larson E, Macauley RJ, Kodl Y, VanDeBogert B, Baawo S, Kruk ME: **Beyond the crisis: did the Ebola epidemic improve resilience of Liberia's health system?** *Health policy and planning* 2017, **32**(suppl_3):iii40-iii47.

62. Mukherjee A, Parashar R: **Impact of the COVID-19 pandemic on the human resources for health in India and key policy areas to build a resilient health workforce**. *Gates open research* 2020, **4**:159.

63. Rawat A, Karlstrom J, Ameha A, Oulare M, Omer MD, Desta HH, Bahuguna S, Hsu K, Miller NP, Bati GT *et al*: **The contribution of community health systems to resilience: Case study of the response to the drought in Ethiopia**. *J Glob Health* 2022, **12**:14001.

64. Tumusiime P, Karamagi H, Titi-Ofei R, Amri M, Seydi ABW, Kipruto H, Droti B, Zombre S, Yoti Z, Zawaira F *et al*: **Building health system resilience in the context of primary health care revitalization for attainment of UHC: proceedings from the Fifth Health Sector Directors' Policy and Planning Meeting for the WHO African Region**. *BMC proceedings* 2020, **14**(Suppl 19):16.

65. Zachariah R, Dar Berger S, Thekkur P, Khogali M, Davtyan K, Kumar AMV, Satyanarayana S, Moses F, Aslanyan G, Aseffa A *et al*: **Investing in Operational Research Capacity Building for Front-Line Health Workers Strengthens Countries' Resilience to Tackling the COVID-19 Pandemic**. *Tropical medicine and infectious disease* 2020, **5**(3).
